# Huimin Insurance and unequal economic welfare between urban and rural areas: evidence from Chinese cities

**DOI:** 10.3389/fpubh.2026.1772176

**Published:** 2026-03-31

**Authors:** Junlong Ma, Shiwei Li, Wenlu Xue

**Affiliations:** College of Economics, Sichuan Agricultural University, Chengdu, Sichuan, China

**Keywords:** commercial health insurance, Huimin insurance, multi-level medical insurance, multi-period DID model, urban-rural consumption gap

## Abstract

**Background:**

The popularization and promotion of Huimin Insurance provide new solutions to alleviate the urban-rural consumption gap and unleash consumption vitality, thereby stimulating domestic demand.

**Methods:**

This article is based on panel data from 224 cities in China from 2015 to 2023. It uses a multi-period DID method to examine the impact of Huimin insurance on the urban-rural consumption gap.

**Results:**

The research results indicate that at the macro-level, the implementation of Huimin insurance is significantly correlated with the narrowing of the urban-rural consumption gap, and this result remains significant after a series of robustness tests. Heterogeneity analysis found that this effect is significant in cities with lower levels of economic development, and further strengthens in Huimin insurance products with higher levels of protection and more participating underwriting institutions. The results of the mechanism test show that the promotion of Huimin insurance may affect the preventive savings behavior of rural residents, thereby affecting the urban-rural consumption gap. Further analysis shows that the Huimin insurance is significantly correlated with the improvement of rural residents' consumption level, while its correlation with urban residents' consumption is not significant.

**Conclusion:**

The research conclusion of this article verifies the inclusive nature of Huimin insurance at the macro level, providing empirical evidence for understanding the potential role of Huimin insurance in narrowing the urban-rural consumption gap and promoting consumption equity. It should be pointed out that due to the lack of microdata, research results may be influenced to some extent by ecological fallacies and aggregation biases. The relevant conclusions mainly reflect macro average correlations, and there are certain boundaries in their explanatory range. Based on this, this article believes that it is necessary to further optimize the institutional design and implementation path of Huimin insurance on the existing basis, and fully exert its universal effect.

## 1 Introduction

Consumption represents final demand and serves as a pivotal link and vital engine for facilitating the domestic economic cycle, directly impacting the safeguarding and improvement of people's livelihoods. The 2025 Central Economic Work Conference prioritized expanding domestic demand, emphasizing the need to “vigorously boost consumption, enhance investment efficiency, and comprehensively expand domestic demand.” This underscores the critical importance of stimulating consumption for achieving all-around expansion of domestic demand. However, in the process of vigorously expanding domestic demand, the urban-rural consumption gap remains a prominent structural bottleneck. This gap not only affects social equity and people's wellbeing but also directly constrains the overall rise in China's consumption level and the full release of domestic demand potential (1). Since the reform and opening-up, China's basic medical insurance system has achieved a historic leap from “system-wide coverage” to “universal coverage.” Data indicates that by the end of 2024, approximately 1.327 billion people nationwide were enrolled in basic medical insurance, with the coverage rate stabilizing above 95%.^1^ This has effectively alleviated the difficulties and high costs of medical treatment for urban and rural residents, laying a crucial foundation for unlocking consumption potential and narrowing consumption disparities. However, as a system designed to “cover the basics,” the scope and level of protection provided by basic medical insurance remain relatively limited, leaving residents with a substantial actual medical burden (2). In 2024, out-of-pocket medical expenses accounted for 27.5% of total medical costs for Chinese residents,^2^ exceeding the global average and indicating that personal medical burdens remain high. Commercial health insurance serves as an effective supplement to basic medical insurance, addressing the shortcomings of low coverage levels in the latter. However, structural issues such as severe product homogeneity, inadequate precision in service delivery, and high enrollment thresholds limit its actual coverage, making it difficult to meet residents' multi-tiered and differentiated healthcare needs (3). Particularly in rural areas and among low-income groups, its penetration rate has remained persistently low, further exacerbating the urban-rural imbalance in the healthcare security system. Against this backdrop, the 2020 “Opinions of the Central Committee of the Communist Party of China and the State Council on Deepening the Reform of the Medical Security System” explicitly called for establishing a multi-tiered medical security system that covers all citizens, integrates urban and rural areas, clarifies rights and responsibilities, provides appropriate coverage, and is sustainable. Huimin insurance emerged as a vital component of China's multi-tiered medical security system and has since developed rapidly.

Current research on Huimin insurance remains in its infancy, with limited existing literature primarily focused on operational models, policy positioning, and product design characteristics, while empirical studies are insufficient. Regarding operational models, Jin et al. (4) note that Huimin insurance essentially operates as a public-private partnership, functioning as a commercial health insurance product under the logic of “government guidance and market operation.” Building on this foundation, academic discourse has delved into the government-business relationship within Huimin insurance, yielding two primary viewpoints: Some scholars advocate that Huimin insurance should adhere strictly to market-driven principles, with the government providing only limited policy guidance (5). Others emphasize that Huimin insurance is not purely market-driven commercial insurance; the government should actively participate in supporting it, treating it as an institutional arrangement with quasi-public goods attributes (6). Furthermore, Kang and Lei (7) extend this analysis from the perspective of state responsibility transformation. They identify challenges in traditional government-guided models, such as imbalances between universal coverage and commercial interests, and propose establishing a new collaborative governance paradigm grounded in “state guarantee responsibility.” In terms of policy positioning, Huimin insurance is widely regarded as a crucial supplementary layer between basic medical insurance and purely commercial health insurance (8), characterized by its inclusive features of “low thresholds, low premiums, and high coverage” (9). Regarding product design, existing research predominantly focuses on its structural features of “high deductibles, high coverage limits, and specialized drug directories” (10, 11). Internationally, public-private partnerships have been widely implemented in health insurance, exemplified by Singapore's government-business commercial health insurance, the U.S. Medicare Advantage (MA) program, and Germany's private supplementary insurance (PKV) under statutory health insurance (12–14). In contrast, substantial research exists on the impact of basic medical insurance (15–19) and traditional commercial health insurance (20, 21) on household consumption, which has generated a wealth of research findings. Literature on urban-rural consumption disparities also predominantly examines urbanization (22–24), the digital economy (1, 25), the internet (26), digital inclusive finance (27, 28), broadband cities (29), agricultural insurance (30), and population aging (31, 32). However, there has been no systematic examination of the role of Huimin insurance in alleviating the medical burden in both urban and rural areas and unlocking the consumption potential of rural residents.

**Figure 1:**
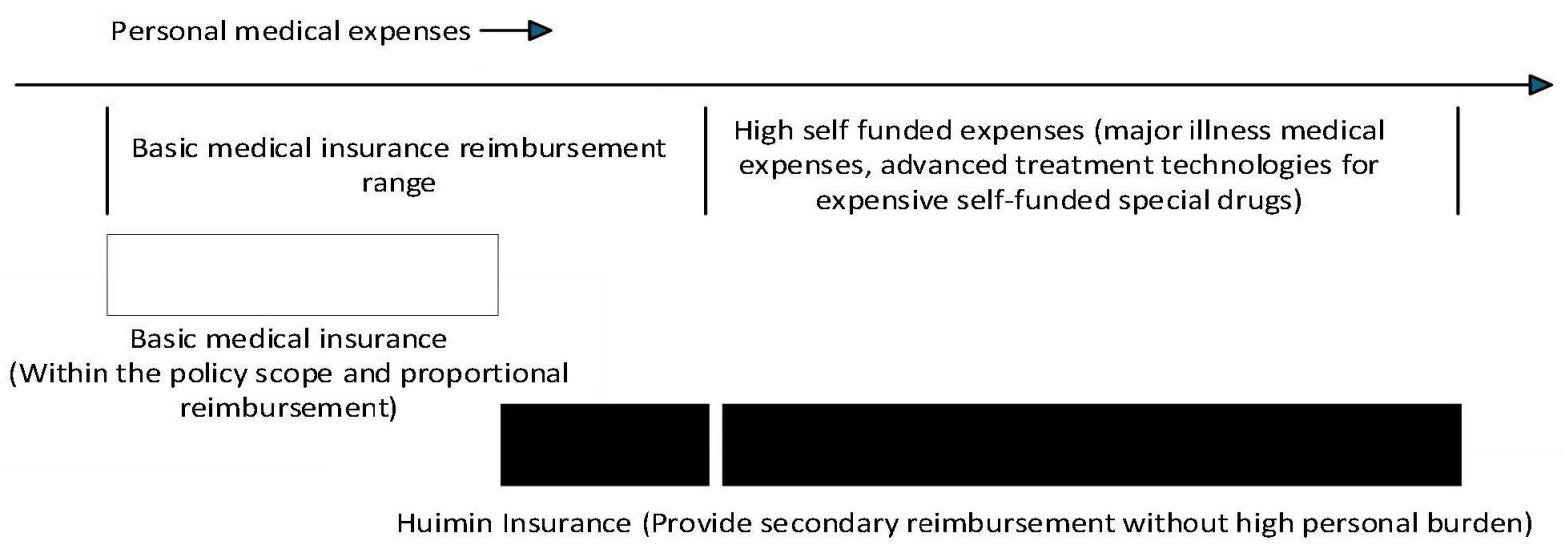
Comparison of Huimin Insurance and basic medical insurance.

It is worth noting that the protection logic of Huimin Insurance is significantly different from that of basic medical insurance. It mainly focuses on high medical expenses and provides secondary supplementary reimbursement for the high out-of-pocket portion that individuals still need to bear after basic medical insurance reimbursement, especially for major illness medical treatment, high-priced self-paid special drugs, and advanced treatment technologies (as shown in Figure 1). This guarantee logic may lead to different pathways and directions of influence in regulating residents' consumption. However, there is currently no systematic research to test the policy effects of Huimin insurance. Therefore, the core issue of this article is whether there is a systematic correlation between Huimin insurance and changes in the urban-rural consumption gap at the macro level, what is its mechanism of action, and whether this effect exhibits regional heterogeneity due to differences in urban customization characteristics. Starting from the perspective of the urban-rural consumption gap, this article provides a good quasi-natural experiment for studying the impact of the policy shock on the urban-rural consumption gap through the gradual promotion process of the pilot program of Huimin Insurance. Based on the panel data of 224 prefecture-level cities in China from 2015 to 2023, a multi-period double difference model is adopted to analyze the correlation between the promotion of Huimin Insurance and the urban-rural consumption gap at the macro level. The heterogeneity of the impact of Huimin Insurance on the urban-rural consumption gap is explored from different perspectives, and further analyze the impact of Huimin Insurance on the consumption of urban and rural residents, respectively. Firstly, in terms of perspective, existing literature mostly focuses on the impact of basic medical insurance or traditional commercial medical insurance on residents' consumption, and there is little research on the relationship between Huimin insurance and the urban-rural consumption gap. This article provides a new research perspective for this field. The second aspect is at the data level. There are relatively few empirical studies on Huimin insurance, which mainly use micro household data. As a customized commercial medical insurance for cities, Huimin Insurance has significant regional characteristics in terms of policy effects. Therefore, microdata is difficult to reflect the reality of implementing policies on a city-by-city basis. This article is based on data from 224 prefecture-level cities in China, which helps to identify the overall effect of Huimin insurance from a macro perspective. Thirdly, at the theoretical level, this article empirically examines the internal transmission mechanism of the impact of Huimin insurance on the urban-rural consumption gap, providing theoretical support for fully utilizing Huimin insurance to stimulate domestic demand and unleash economic growth vitality. This study confirms the inclusive nature of Huimin insurance, which helps enrich the research content of medical insurance and consumption, and provides a scientific theoretical basis and empirical support for effectively exerting the inclusive function of Huimin insurance and alleviating the urban-rural consumption gap.

## 2 Policy context and research hypotheses

### 2.1 Policy context

Huimin Insurance (also known as “Universal Commercial Health Insurance” or “City-Customized Commercial Health Insurance”) is a supplementary health insurance product guided by local governments, underwritten by commercial insurance companies, and targeted at participants in basic medical insurance. In 2016, the “Healthy China 2030” Planning Outline charted the course for commercial health insurance development at the top-level design stage. In 2020, the Central Committee of the Communist Party of China and the State Council issued the “Opinions on Deepening the Reform of the Medical Security System,” emphasizing the need to “establish a multi-tiered medical security system,” which provided fundamental guidance for exploring inclusive health insurance. In 2021, the China Banking and Insurance Regulatory Commission (CBIRC) issued the first dedicated document, the “Notice on Regulating Insurance Companies' City-Customized Commercial Medical Insurance Business,” establishing the fundamental principle of “government guidance and market operation.” It also emphasized for the first time that while supporting development, risks must be guarded against and commercial sustainability upheld. This marked the transition of Huimin insurance development from spontaneous local exploration to a new era of standardized national guidance. In October 2023 and January 2024, the National Financial Regulatory Administration successively drafted two versions of the “Notice on the Smooth and Orderly Implementation of City-Customized Commercial Medical Insurance (Draft for Comment).” While reaffirming the core principle of market-oriented operation, these documents explicitly positioned Huimin Insurance as a commercial market activity, further clarifying the boundaries between government and market. This signaled a shift in the government's role from “front-end dominance” to “back-end guidance.” In 2024, the National Financial Regulatory Administration released the “Guiding Opinions on Promoting High-Quality Development of Inclusive Insurance,” systematically proposing four principles: accessibility, affordability, protective attributes, and sustainability. This established a more robust institutional framework for the long-term healthy development of Huimin insurance. In 2025, the National Financial Regulatory Administration issued the “Notice on Promoting High-Quality Development of Urban Commercial Medical Insurance,” further clarifying the responsibilities between the government and the market. For the first time, it explicitly required “differentiated pricing” based on age, gender, and health status, while strictly prohibiting “low-price, cutthroat competition” and pre-setting claim ratios. This marked the formal transition of Huimin Insurance from extensive expansion to a phase of refined, sustainable, high-quality development.

**Figure 2:**
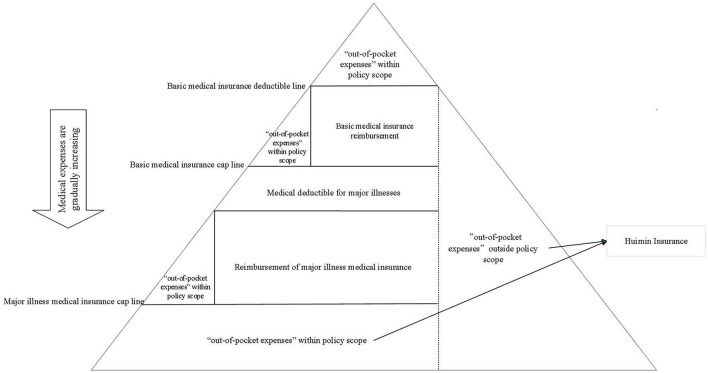
Scope of coverage for each medical insurance.

The emergence and development of Huimin insurance stem from the continuous refinement of China's multi-tiered medical security system and the existing gaps in healthcare coverage. China has preliminarily established a multi-tiered medical security framework centered on basic medical insurance as the foundation, with medical assistance serving as the safety net, and supplemented by the coordinated development of supplementary medical insurance and commercial health insurance. However, practical implementation still reveals certain coverage gaps: as shown in Figure 2, basic medical insurance imposes a “deductible threshold” and a “coverage cap,” reimbursing only a percentage of medical expenses within the policy scope. Costs below the deductible threshold and above the coverage cap must be borne entirely by the individual. When these expenses exceed the “major illness deductible,” they become eligible for reimbursement under major illness insurance, though individuals still bear a portion of the costs after reimbursement. If medical expenses further exceed the “major illness insurance cap,” the excess amount is entirely borne by the individual. Additionally, the basic medical insurance system typically does not cover medications, diagnostic procedures, or service facilities outside the medical insurance catalog, resulting in “out-of-pocket” expenses. To address these gaps, traditional commercial health insurance can provide some supplementation. However, its high premiums and stringent health underwriting conditions often exclude large numbers of older adults and those with pre-existing conditions from coverage. Against this backdrop, Huimin Insurance emerged as a type of inclusive commercial health insurance. Its coverage extends to both “out-of-pocket expenses” within policy scope and “personal out-of-pocket expenses” outside policy scope. By setting reasonable annual cumulative deductibles and tiered reimbursement ratios, it prioritizes compensation for high medical expenditures, effectively enhancing residents' economic resilience against critical illness risks. Its core characteristics encompass three aspects: First, inclusivity and broad coverage, enabling vulnerable groups and older individuals to access supplemental medical protection through reduced premiums and relaxed eligibility requirements (33). Second, customization and regional adaptability, leveraging local government participation in product design and promotion to develop locally tailored “one city, one policy” solutions (34). Third, public-private partnerships and market-oriented operations, adhering to principles of equal opportunity, non-discrimination, and commercial sustainability under a “government-guided, market-operated” framework (35). Historically, the system originated from Shenzhen's 2015 “Supplementary Medical Insurance for Critical Illnesses.” The period from 2015 to 2019 served as a policy pilot phase, during which only four new products were introduced nationwide: Nanjing's “Hui Min Health Insurance” in 2018, Foshan's “Foshan Medical Insurance” in 2019, Guangzhou's “Guangzhou Hui Min Insurance,” and Zhuhai's “Great Love Without Borders.” Development progressed relatively slowly. In 2020, with government support, Huimin Insurance entered a phase of explosive growth, forming a collaborative model involving “government departments + insurance companies + third-party platforms.” Data from the 2024 Knowledge Map of City-Customized Commercial Medical Insurance (Hui Min Insurance) indicates that as of October 31, 2024, 298 products had been launched nationwide, covering all 31 provincial-level administrative regions. Municipal-level products accounted for over 80% of the total, reflecting a tiered development pattern characterized by “provincial coordination and municipal leadership.” However, the sustainable development of Huimin Insurance still faces multiple challenges. At the operational level, adverse selection risks are prominent, market irregularities frequently occur, and data silos remain unresolved (36). At the institutional level, inherent tensions are increasingly evident—functioning as market-based commercial products while fulfilling social functions perpetuates the risk of a “death spiral” (37). Regarding developmental effectiveness, the current overall enrollment scale remains insufficient compared to basic medical insurance and traditional commercial health insurance, limiting the full realization of its inclusive benefits (38).

### 2.2 Research hypotheses

#### 2.2.1 The direct impact of Huimin Insurance on the urban-rural consumption gap

According to consumer theory and risk-sharing theory, when households face uncertainty regarding health risks and medical expenditures, their consumption decisions are constrained not only by current income but also by the distribution of potential losses and the degree of their volatility (39, 40). Health insurance can mitigate consumption disparities across different groups by enhancing risk protection levels, altering households' optimal consumption choices, and enabling higher and more stable consumption paths under identical income conditions (41–43). Specifically, within China's urban-rural dual structure, rural residents have long endured a “tight constraint” state characterized by relatively inadequate healthcare coverage and scarce risk-hedging tools, facing elevated medical risks and stringent intertemporal budget constraints (44, 45). The Huimin insurance, characterized by “low thresholds and high coverage,” provides rural households with effective protection against tail risks such as critical illnesses and severe conditions, boosting their consumption confidence and stimulating current consumption growth. Meanwhile, since urban residents already enjoy relatively comprehensive healthcare coverage, the marginal utility gains from such insurance are less pronounced for them but particularly significant for rural residents (46). Therefore, by enhancing rural residents' ability to smooth consumption across periods, Huimin insurance drives greater consumption growth among rural populations than urban residents. This dynamic process gradually narrows the urban-rural consumption gap. In summary, this article proposes the following assumptions:

H1: Huimin insurance can narrow the urban-rural consumption gap.

#### 2.2.2 Analysis of the mechanism by which Huimin Insurance affects the urban-rural consumption gap

The precautionary savings theory posits that risk-averse individuals, faced with future uncertainty, will increase savings and reduce current consumption to hedge against potential negative shocks (47). Compared to urban residents, rural residents confront a more fragile social security system and higher exposure to health risks, resulting in particularly strong precautionary savings motives (48, 49). The core mechanism by which Huimin insurance mitigates urban-rural consumption disparities lies in its ability to more effectively alleviate rural residents' precautionary savings motivation, thereby reducing their precautionary savings (46). Huimin Insurance alleviates rural residents' precautionary savings primarily through three mechanisms. First, it reduces uncertainty in medical expenditures. Rural households possess weak financial assets and poor risk resilience, making them highly sensitive to the steep out-of-pocket costs associated with major illnesses (50). This fear of “falling into poverty due to illness” constitutes a significant psychological driver of intense precautionary savings. As an effective supplement to basic medical insurance, Huimin Insurance significantly reduces the probability of catastrophic medical expenditures by signaling stable policy safety nets and focusing on reimbursement for high medical costs (51). When rural residents perceive their health risks are effectively covered, their concerns about future financial risks diminish substantially, enabling them to convert more savings into consumption (52, 53). Second, it enhances rural residents' consumption confidence and marginal propensity to consume. Existing research indicates that health insurance can improve residents' risk expectations and increase their marginal propensity to consume (54, 55). While strengthening rural residents' resilience against health risks, Huimin insurance also bolsters their confidence in their own medical and health security. With reduced perceived risk, rural residents will gradually decrease the proportion of precautionary savings in their budget allocation. Finally, it bridges the urban-rural gap in medical security. Under the urban-rural dual structure, rural residents have long been disadvantaged in accessing public medical services and social security (56). The promotion of Huimin insurance partially bridges this institutional gap in healthcare coverage, narrowing the “security gap” residents face when confronting health risks. This, in turn, alleviates the increased precautionary savings stemming from unequal protection, unleashes their consumption potential, and reduces the urban-rural consumption gap. Therefore, based on the above, this paper proposes the following hypothesis:

H2: Huimin insurance can reduce the urban-rural consumption gap by lowering the amount of precautionary savings among rural residents.

## 3 Research design

### 3.1 Model design

This study employs a Multi-Period difference-in-differences (DID) model to evaluate the impact of Huimin insurance on the urban-rural consumption gap. The primary reason for selecting this model is that the Huimin insurance program was rolled out incrementally across cities, with different cities implementing it at varying times. Furthermore, the Multi-Period DID model effectively addresses the limitation of traditional DID models, which are constrained by a single policy implementation time point. By utilizing city-level panel data, it captures the dynamic effects of implementing Huimin insurance across different cities at different time points. The specific model is as follows:


$$\begin{array}{l} Gap_{it}=\alpha +\beta Treat_{i}\times Time_{t}+\gamma Z_{it}+\varphi_{i}+\mu_{t}+\varepsilon_{it} \end{array}$$ (1)

The dependent variables *Gap*_*it*_ represent the urban-rural consumption gap for city i in year t; *Treat*_*i*_ denotes the dummy variable for pilot cities, *Time*_*t*_ represents the dummy variable for the period before and after the pilot program, and the interaction term *Treat*_*i*_×*Time*_*t*_ (*did*) serves as the core explanatory variable in this study; *Z*_*it*_ indicates the control variables affecting the urban-rural consumption gap; φ_*i*_ and μ_*t*_ denote the city and year fixed effects, respectively; ϵ_*it*_ is the random disturbance term; β is the estimator measuring the impact of the on the urban-rural consumption gap.

Based on the preceding theoretical analysis, Huimin Insurance primarily narrows the urban-rural consumption gap by reducing rural residents' precautionary savings. To test this mechanism, this paper draws on Jiang's (57) approach and constructs the following model for verification:


$$\begin{array}{l} M_{it}=\alpha_{1}+\beta_{1}Treat_{i}\times Time_{t}+\gamma_{1}Z_{it}+\varphi_{i}+\mu_{t}+\varepsilon_{it} \end{array}$$ (2)

Where *M*_*it*_ in Equation 2 denotes the mechanism variable representing the precautionary savings of rural residents in city i during year t, with the remaining variables consistent with Equation 1.

### 3.2 Variable selection

#### 3.2.1 Dependent variable

The dependent variable in this study is the urban-rural consumption gap, measured by the ratio of per capita consumption expenditure among urban residents to that among rural residents.

#### 3.2.2 Core explanatory variable

The core explanatory variable of this article is the Huimin Insurance (*did*). It is a policy dummy variable. Considering that the timing of the introduction of Huimin insurance varies among cities, most of them were launched after the outbreak of the pandemic in 2020, and some cities have introduced several different types of Huimin insurance. Meanwhile, considering that some provinces' provincial-level comprehensive Huimin insurance products were launched earlier than their prefecture-level cities' city-customized Huimin insurance products, such as Henan Province's “Zhongyuan Medical Huimin Insurance,” launched in 2020, and Guangxi's “Huigui Insurance.” Therefore, this article is based on the launch time of the earliest coverage of Huimin insurance products in the region. Among them, the construction of *did* is as follows: *Treat*_*i*_ is the virtual variable of the treatment group. If there are Huimin insurance products in city i, *Treat*_*i*_is taken as 1; otherwise, it is taken as 0. *Time*_*t*_ is a dummy variable for policy implementation time. If a city launches a Huimin insurance product after t years, it takes 1 in t years and beyond, otherwise it takes 0.

#### 3.2.3 Control variable

In order to control the impact of other factors on the urban-rural consumption gap, this paper selects certain control variables from different dimensions, including the level of economic development (*Ln*_*per*_*gdp*), fiscal expenditure (*Ln*_*gov*), education expenditure (*Education*), digital inclusive financial index (*Finance*), medical level (*Medical*), basic pension insurance for employees (*Pen*), basic medical insurance for employees (*Med*), industrial structure (*Ind*) and Internet penetration (*Inter*). Detailed variable descriptions can be found in the descriptive statistics of Table 1.

**Table 1:** Descriptive statistics.

**Variable name**	**Variable symbol**	**Variable definition**	***N***	**Mean**	**SD**	**Min**	**Max**
Urban-rural consumption gap	Gap	Per capita consumption expenditure ratio of urban and rural residents	2016	1.835	0.325	1.117	4.158
Urban residents' consumption	Ln_u_i	Ln(per capita consumption expenditure of urban residents)	2016	10.050	0.273	9.321	10.914
Rural residents' consumption	Ln_r_i	Ln(per capita consumption expenditure of rural residents)	2016	9.458	0.335	8.336	10.467
Huimin insurance	did	Policy dummy variable	2016	0.375	0.484	0.000	1.000
Economic development level	Ln_per_gdp	Ln(GDP per capita)	2016	10.987	0.508	9.688	12.207
Fiscal expenditure	Ln_gov	Ln(expenditure within the general budget of local finance)	2016	15.253	0.702	13.493	17.787
Education expenditure	Education	Education expenditure/expenditure within the general budget of local finance	2016	0.175	0.033	0.073	0.304
Digital inclusive finance	Finance	Digital inclusive finance index of prefecture-level cities	2016	248.002	47.040	140.961	373.222
Medical level	Medical	Number of beds per 100 people in hospitals and health centers	2016	0.499	0.178	0.183	1.111
Employee basic pension insurance coverage rate	Pen	Number of employees participating in basic pension insurance for employees/total population	2016	0.196	0.129	0.045	0.679
Employee basic medical insurance coverage rate	Med	Number of employees participating in basic medical insurance for employees/total population	2016	0.241	0.132	0.028	0.819
Industrial structure	Ind	Value added of the tertiary industry/value added of the secondary industry	2016	1.204	0.569	0.333	4.800
Internet popularity	Inter	Number of internet broadband subscribers per 100 people	2016	33.329	17.289	5.289	94.692

### 3.3 Data source

This article selects the period from 2015 to 2023 as the research interval. Due to the lack of key variable data in some cities, 224 cities were ultimately selected as the research sample. The relevant data of Huimin Insurance comes from the manual sorting of the information of Huimin Insurance published by the official account and insurance application applet of the relevant government departments at and above the municipal level. The rest of the data are mainly from the China Urban Statistical Yearbook over the years. For some missing values, this article manually fills in and linearly interpolates them based on the national economic and social development statistical bulletins of each prefecture-level city for the current year. The descriptive statistical results of each variable are shown in Table 1.

## 4 Empirical result analysis

### 4.1 Benchmark regression

The estimation results of model (1) are listed in Table 2, where columns (1) and (2) control for fixed effects of city and year, but column (1) does not include control variables, while column (2) includes control variables. It can be seen that, regardless of whether control variables are added or not, the coefficients of the core explanatory variable are significantly negative. Hypothesis H1 has been verified.

**Table 2:** Benchmark regression results.

**Variables**	**(1) Gap**	**(2) Gap**
DID	−0.034^**^ (−2.290)	−0.040^***^ (−2.619)
Ln_per_gdp		−0.070^**^ (−2.192)
Ln_gov		0.048 (1.034)
Education		0.369 (1.253)
Finance		−0.002^**^ (−2.077)
Medical		−0.026 (−0.297)
Pen		0.297^***^ (2.744)
Med		−0.008 (−0.092)
Ind		−0.115^***^ (−6.986)
Inter		−0.000 (−0.226)
Constant	2.000^***^ (217.532)	2.313^***^ (3.106)
City FE	Yes	Yes
Time FE	Yes	Yes
Observation	2016.000	2016.000
*r* ^2^	0.484	0.501

^***^, ^**^, and ^*^ represent significance levels of 1%, 5%, and 10%, respectively, with T-values in parentheses.

### 4.2 Parallel trend test

**Figure 3:**
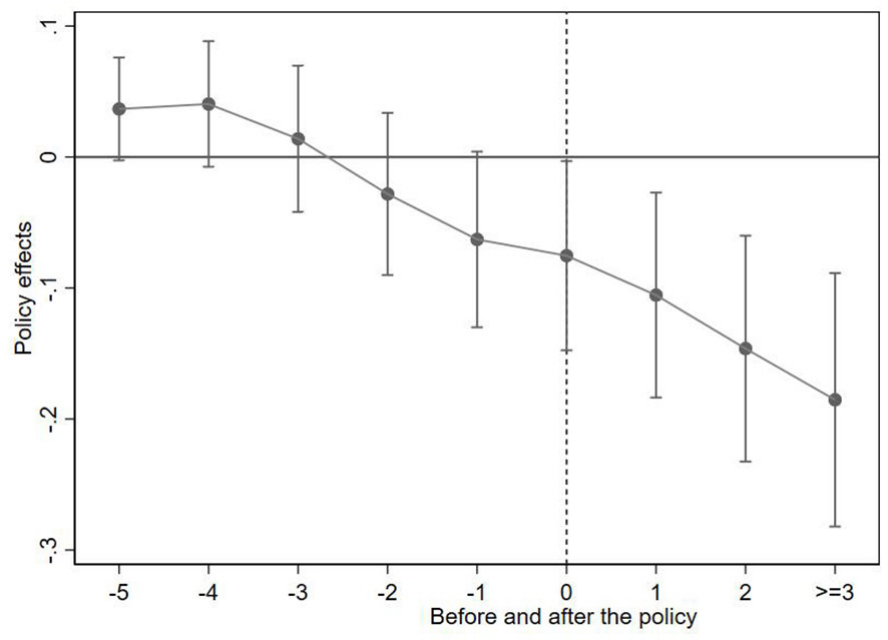
Parallel trend test.

The prerequisite for evaluating the impact of implementing Huimin insurance in cities on the urban-rural consumption gap is to conduct parallel trend tests on policy shocks. If there is no significant difference in the urban-rural consumption gap between the treatment group and the control group before the policy shock, it indicates that the parallel trend test has passed. Considering that there were relatively few cities that introduced Huimin insurance before 2020, and that Huimin insurance experienced explosive growth in 2020, this article sets 2020 as the time for policy shocks. Due to the different years of introduction of Huimin Insurance in different cities, the relative time span of event research presents as eight periods before and five periods after the outbreak of Huimin Insurance growth. Through observation, it was found that there were fewer samples in the seventh and eighth periods before the outbreak of Huimin Insurance growth, as well as in the fourth and fifth periods after. To avoid estimation instability caused by sparse tail samples, this article summarizes the samples from the seventh and eighth periods before policy implementation into −6 periods, and the samples from the fourth and fifth periods after policy implementation into 3 periods. In terms of specific methods, this article draws on the approach of Beck et al. (58) by calculating the prior mean and adjusting all regression coefficients and confidence intervals by removing the mean to control for possible prior trends. In addition, this article follows the approach of Wang and Ge (59) and uses the sixth period (i.e., −6 periods) before policy implementation as the base period. Based on the above ideas and methods, process the article data and draw a parallel trend chart. As shown in Figure 3, the estimated coefficients for each period before policy implementation are close to zero, and their 95% confidence intervals all contain zero values. This indicates that there is no significant trend difference between the treatment group and the control group before policy intervention, satisfying the parallel trend hypothesis.

### 4.3 Endogenous treatment

Considering that the introduction of Huimin insurance in prefecture-level cities is not a random occurrence, but depends on local economic development level, population structure, fiscal capacity, and governance capacity, the benchmark DID estimation in this article may have a selective bias. At the same time, cities with smaller urban-rural consumption gaps and higher levels of economic integration may have stronger residents' ability and demand for supplementary medical insurance, and local governments may also introduce Huimin insurance due to increased demand for public services, leading to potential reverse causality. Therefore, there may be endogeneity issues between the two, and this article further uses the instrumental variable method and the PSM-DID method for robustness testing.

#### 4.3.1 Instrumental variables method

This article refers to the approach of Liu et al. (60) in constructing instrumental variables, selecting the number of cities in the same province that have covered Huimin insurance in the past year, except for this city, as the instrumental variable (IV). Drawing on the approach of Tian and Zhang (61), the interaction term of IV ^*^ Post is constructed as the core explanatory variable as the instrumental variable, and two-stage instrumental variable tests are conducted. Table 3, column (1) reports the estimation results of the first stage. It can be seen that the coefficient of the interaction term is significantly positive, and the F-statistic is 221.274, far greater than the critical value of 10, indicating that the instrumental variables satisfy the correlation characteristics and there is no weak instrumental variable problem. Column (2) includes the core explanatory variable and the interaction term as explanatory variables in the regression. It can be found that the coefficient of the core explanatory variable is still significantly negative, and the coefficient of the interaction term is not significant, indicating that the instrumental variable satisfies the exclusivity constraint. Column (3) shows the estimated results of the second stage. It can be seen that the Huimin insurance can still effectively promote the narrowing of the urban-rural consumption gap, further confirming the robustness of the benchmark results.

**Table 3:** Endogenous processing results.

**Variables**	**(1) First Stage**	**(2) Include instrumental variables**	**(3) Second stage**	**(4) Neighbor matching**	**(5) Caliper matching**	**(6) Nuclear matching**
	**Did**	**Gap**	**Gap**	**Gap**	**Gap**	**Gap**
DID		−0.041^***^ (−2.622)	−0.205^**^ (−2.378)	−0.055^***^ (−3.027)	−0.108^***^ (−4.494)	−0.090^***^ (−3.814)
IV^*^Post	0.037^***^ (14.876)	0.001 (0.389)				
Cragg–Donald Wald F statistic			221.274			
Control	Yes	Yes	Yes	Yes	Yes	Yes
City FE	Yes	Yes	Yes	Yes	Yes	Yes
Time FE	Yes	Yes	Yes	Yes	Yes	Yes
Constant	−0.785^***^ (−3.539)	2.322^***^ (3.116)	1.604^***^ (5.502)	2.644^***^ (2.681)	2.787^*^ (1.822)	3.313^***^ (2.249)
Observation	2016.000	2016.000	2016.000	1598.000	785.000	863.000
*r* ^2^	0.786	0.501	0.212	0.495	0.503	0.497

^***^, ^**^, and ^*^ represent significance levels of 1%, 5%, and 10%, respectively, with T-values in parentheses.

#### 4.3.2 PSM-DID method

This article uses all control variables in the benchmark regression as covariates, and calculates propensity score using nearest neighbor one-to-one matching, caliper matching, and kernel matching. Logistic regression is performed on cities that have implemented Huimin insurance to find cities with similar propensity scores. After screening the sample, regression is performed again, and the results are shown in columns (4), (5), and (6) of Table 3. The coefficients of the core explanatory variables are significantly negative, which is consistent with the benchmark results of this article.

### 4.4 Robustness test

#### 4.4.1 Placebo test

**Figure 4:**
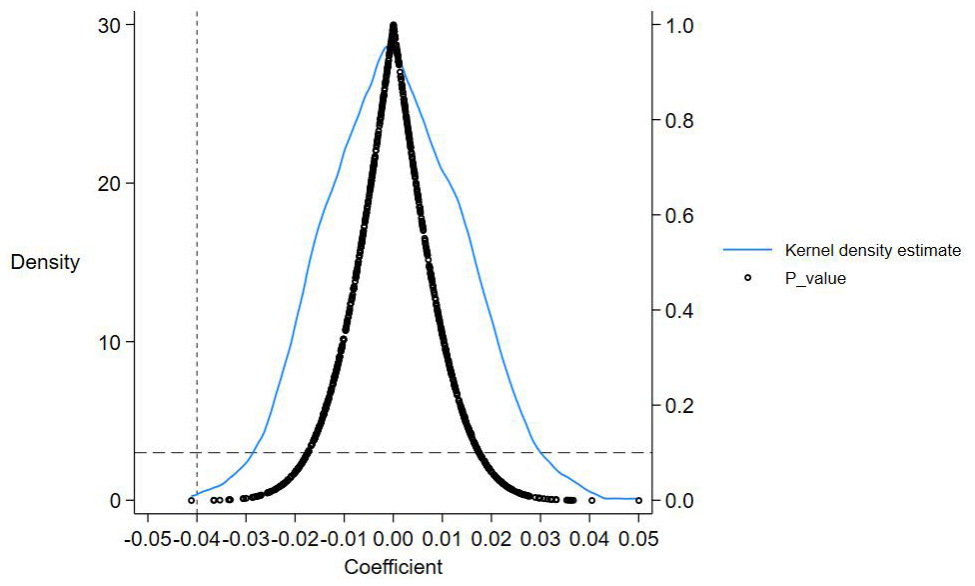
Placebo test.

In order to avoid the influence of non-observed urban characteristics on the results, this study conducted a placebo test on the regression results. Following the approach of Bai et al. (62), the city treatment group and policy implementation time for implementing Huimin insurance were randomized. The specific method is to select cities with the same number of implemented policies from all city samples as the treatment group dummy variable, and randomly select the sample period of policy impact time for the implemented cities as the policy time dummy variable. This article maintains the same size of the treatment group, conducting 1,000 random samples, and reports the distribution and *p*-value of the estimated coefficients of the regression results in Figure 4. From Figure 4, it can be seen that the coefficients of the fictional processing effect are mostly distributed near zero, showing a typical normal distribution centered around zero, and the *p*-values are mostly greater than 0.1. At the same time, the real coefficient represented by the dashed line is −0.40, significantly deviating from the distribution range of the estimated coefficients of the random sample, indicating that the placebo test passed.

#### 4.4.2 Heterogeneity processing effect test

Given that there may be bias in estimating the two-way fixed effects (TWFE) estimator in multi- period DID models. In this regard, this article draws on the multi-period dual robust estimator (Callaway and Sant'Anna, CSDID) method proposed by Callaowaw and Sant'Anna (63) for further robustness testing. This method is based on the idea of dual robustness, which helps to avoid the problem of traditional DID estimation bias. Its core idea is to divide the sample into different subgroups, estimate the treatment effects of different groups separately, and then use specific strategies to sum up the treatment effects of different groups to calculate the average treatment effect (ATT) of the sample period. The principle of the aggregation strategy is to reduce the aggregation weight of groups that may have bias. The estimated results are shown in columns (1)–(4) of Table 4. It can be found that the average treatment effects of the four different types all indicate that the Huimin insurance can reduce the urban-rural consumption gap, which is consistent with the benchmark results, indicating that this conclusion is robust.

**Table 4:** Robustness test 1.

**Variables**	**Simple weighted average processing effect**	**Dynamic average processing effect**	**Calendar average processing effect**	**Group average processing effect**
	**(1)**	**(2)**	**(3)**	**(4)**
Simple ATT	−0.047^***^ (−2.982)			
Pre_avg		0.033 (0.907)		
Post_avg		−0.066^**^ (−1.978)		
CAverage			−0.048^***^ (−4.047)	
CAverage				−0.040^***^ (−2.685)

^***^, ^**^, and ^*^ represent significance levels of 1%, 5%, and 10%, respectively, with T-values in parentheses.

#### 4.4.3 Replace the explained variable

Considering that the urban-rural gap mainly manifests as changes at both ends, and the Theil index is more sensitive to changes at both ends, directly taking the ratio will not be sensitive to the actual gap and may cause bias in the process of measuring inequality. Therefore, this article uses the Theil index of urban and rural residents' consumption as a replacement dependent variable to conduct a robustness test again. As shown in column (1) of Table 5, it can be observed that the coefficient of the core explanatory variable remains significantly negative.

**Table 5:** Robustness test 2.

**Variables**	**(1) Replace variables**	**(2) Exclude samples**	**(3) Interactive fixed effect**	**(4) Time trend**
	**Theil**	**Gap**	**Gap**	**Gap**
DID	−0.003^*^ (−1.914)	−0.046^***^ (−3.002)	−0.025^*^ (−1.725)	−0.040^***^ (−2.622)
Control	YES	YES	YES	YES
City FE	YES	YES	YES	YES
Time FE	YES	YES	NO	YES
Time × Province FE	NO	NO	YES	NO
Constant	0.141^*^ (1.953)	1.930^**^ (2.499)	2.296^***^ (2.7336)	2.299^***^ (3.095)
Observation	2016.000	1953.000	1962.000	2016.000
*r* ^2^	0.371	0.499	0.018	0.501

^***^, ^**^, and ^*^ represent significance levels of 1%, 5%, and 10%, respectively, with T-values in parentheses.

#### 4.4.4 Exclude samples

Considering the special administrative status of municipalities directly under the central government and municipalities separately listed in the sample, this article conducts robustness tests after removing them from the sample. Table 5, column (2) reports the estimated results, indicating that after excluding samples from municipalities directly under the central government and individual municipalities, the coefficient of the core explanatory variable is significantly negative, consistent with the baseline results of this study.

#### 4.4.5 Interactive fixed effect

Given that the policy effects of Huimin Insurance may be affected by policy factors at the provincial level during the same period, such as central government transfer payments and other policy arrangements implemented uniformly by the provincial level, this article further introduces the interactive fixed effects between provinces and years based on the benchmark model. The estimated results are reported in column (3) of Table 5. The results showed that after incorporating the fixed effects of interaction between provinces and years, the coefficient of the core explanatory variable remained significantly negative, consistent with the baseline regression, indicating that the main conclusions of this study are still robust.

#### 4.4.6 Control non-parallel trend terms

To eliminate estimation errors caused by the time trend of control variables in the model. This article follows the approach of Sun et al. (64) and adds an interaction term between time trend and control variables in (1). The estimated results are shown in column (4) of Table 5. It can be seen that after controlling for the time trend, the coefficient of the core explanatory variable is still significantly negative.

#### 4.4.7 Eliminate policy interference

To accurately identify the net policy effect of Huimin insurance, it is necessary to control for other policies that may affect the urban-rural consumption gap during the sample period. Considering that the sample study period is from 2015 to 2023. Therefore, this article incorporates four policies into the model: the “Broadband China” demonstration city implemented in 2014 (*Broadband*_*China*), the “Poverty Alleviation” policy for poverty-stricken counties at the national level determined in 2015 (*Poverty*_*Alleviation*), the “New Urbanization” comprehensive pilot area (*New*_*Urbanization*), and the Integrate the basic medical insurance system for urban and rural residents implemented in 2016 (*Together*). Regarding the targeted poverty alleviation policy, given that 2020 is both the final year of comprehensive victory in poverty alleviation and the explosive growth period of Huimin insurance, the “Opinions of the Central Committee of the Communist Party of China and the State Council on Effectively Linking Consolidating and Expanding the Achievements of Poverty Alleviation with Rural Revitalization” also points out that after the completion of poverty alleviation goals and tasks, a five-year transition period will be established, and the policy focus will shift from centralized poverty alleviation to consolidating achievements and rural revitalization. Therefore, the policy effect of Huimin insurance is mainly affected by the sustained effect of the “post-poverty alleviation era.” Therefore, this article takes 2020 as the phased boundary point for targeted poverty alleviation policies. Specifically, this article characterizes the degree of poverty alleviation policy exposure at the urban level by the number of national poverty-stricken counties within the jurisdiction of prefecture-level cities, and constructs a treatment group based on this. At the same time, 2020 is used as the standard for dividing the time dummy variable (Post). The construction method of other policy variables is similar to that of Huimin insurance, both of which are the interaction terms of policy processing variables and time variables. The regression results are shown in Table 6. Columns (1)–(4) report the regression results for each policy included separately, while column (5) controls for all four policies simultaneously. It can be seen that the coefficients of the core explanatory variables are significantly negative, further confirming the robustness of the benchmark results.

**Table 6:** Robustness test 3.

**Variables**	**(1) Targeted poverty alleviation**	**(2) Medical insurance integration**	**(3) Urbanization**	**(4) Broadband China**	**(5) All policies**
	**Gap**	**Gap**	**Gap**	**Gap**	**Gap**
DID	−0.042^***^ (−2.819)	−0.042^***^ (−2.751)	−0.039^***^ (−2.612)	−0.039^***^ (−2.597)	−0.044^***^ (−2.919)
Poverty_Alleviation	−0.009^***^ (−3.960)				−0.009^***^ (−3.094)
Together		−0.025^**^ (−2.050)			−0.025^**^ (−2.035)
New_Urbanization			−0.015 (−0.412)		−0.021 (−0.576)
Broadband_China				0.031 (1.187)	0.028 (1.069)
Control	YES	YES	YES	YES	YES
City FE	YES	YES	YES	YES	YES
Time FE	YES	YES	YES	YES	YES
Constant	2.177^***^ (2.933)	2.288^***^ (3.076)	2.317^***^ (3.110)	2.333^***^ (3.133)	2.179^***^ (2.936)
Observation	2016.000	2016.000	2016.000	2016.000	2016.000
*r* ^2^	0.506	0.502	0.501	0.502	0.507

^***^, ^**^, and ^*^ represent significance levels of 1%, 5%, and 10%, respectively, with T-values in parentheses.

### 4.5 Heterogeneity analysis

#### 4.5.1 Heterogeneity analysis of guarantee level

The level of protection is a key factor affecting residents' decision-making on insurance participation (65), and its differences not only directly affect their willingness to participate in insurance but also determine the actual effectiveness of insurance policies in achieving social welfare and redistribution functions. To examine whether the impact of Huimin insurance on urban-rural consumption disparities exhibits heterogeneity across different coverage levels, this study constructs a comprehensive evaluation system for coverage levels based on three dimensions: coverage breadth, coverage depth, and preferentiality. The specific indicator construction methods are detailed in Table 7. To ensure the robustness of the indicator system, two approaches are employed to construct the coverage level indicators. Based on this, the interaction terms between the core explanatory variable and coverage level coefficients were constructed for testing (*did*_*Coverage*1 and *did*_*Coverage*2). Results in Column (1) of Table 8 show that the interaction coefficients from both methods are significantly negative, indicating that Huimin insurance with higher coverage levels exerts a stronger mitigating effect on the urban-rural consumption gap. A possible reason lies in the fact that more comprehensive Huimin insurance offers greater coverage in terms of benefit limits, reimbursement rates, and scope of liability. It provides stronger risk mitigation for critical illnesses and high medical expenses, thereby more effectively alleviating the uncertainty of healthcare costs and tail risks faced by households (66). Compared to urban residents, rural residents have lower income levels, relatively weaker medical coverage, and greater exposure to sudden medical expenditure risks. Their consumption decisions are more sensitive to changes in medical risks (67). Therefore, Huimin insurance with higher coverage levels can significantly reduce rural households' propensity for precautionary savings, unleash their consumption demand, and thereby more effectively narrow the urban-rural consumption gap.

**Table 7:** Guarantee level indicator system.

**First–level indicator**	**Secondary indicator**	**Computational method**	**Indicator attribute**	**Method 1 weight**	**Method 2 weight**
Protection breadth	Number of contents protected by Huimin Insurance	Actual guarantee quantity/maximum guarantee quantity	Positive	0.4	0.45
Protection depth^a^	Reimbursement ratio of hospitalization expenses within the scope of medical insurance	(Actual reimbursement ratio – Minimum ratio)/(Maximum ratio – Minimum ratio)	Positive	0.4	0.45
	Deductible amount for personal burden within the scope of medical insurance	1- (Actual deductible – Minimum deductible)/(Maximum deductible – Minimum deductible)	Positive		
Preferential treatment	Does it cover high-priced treatment technologies (such as proton, heavy ion, and CAR-T therapy) and rare diseases?	Actual coverage content/2	Positive	0.2	0.1

^a^The formula for calculating the depth of protection is: (score of hospitalization expense reimbursement ratio within the scope of medical insurance+score of personal burden deductible within the scope of medical insurance)/2.

**Table 8:** Heterogeneity analysis 1.

**Variables**	**(1) Guarantee level**		**(2) Underwriting companies**
	**Gap**	**Gap**	**Gap**
did_Coverage1	−0.079^**^ (−2.326)		
did_Coverage2		−0.086^***^ (−2.711)	
did_joi			−0.008^***^ (−4.884)
Control	YES	YES	YES
City FE	YES	YES	YES
Time FE	YES	YES	YES
Constant	2.397^***^ (3.227)	2.381^***^ (3.206)	2.535^***^ (3.434)
Observation	2016.000	2016.000	2016.000
*r* ^2^	0.501	0.501	0.506

^***^, ^**^, and ^*^ represent significance levels of 1%, 5%, and 10%, respectively, with T-values in parentheses.

#### 4.5.2 Heterogeneity analysis of different numbers of underwriting companies

Consumers' purchasing decisions for insurance depend on their perception of provider reliability and contract durability (68). When there are a large number of underwriting companies for a certain insurance product, it often enhances consumers' trust in the stability of the insurance, thereby increasing their willingness to participate in insurance. This behavioral change not only affects the coverage effect of insurance itself, but also changes the level of protection and consumption expectations of residents, ultimately affecting the overall welfare level and policy effectiveness of this insurance (69). Therefore, it can be inferred that the difference in the number of underwriting companies in inclusive insurance, such as Huimin Insurance, is likely to affect consumers' participation decisions, thereby affecting the urban-rural consumption gap in the region. To test this inference, this article constructs a policy dummy variable and the interaction term between the number of underwriting companies (*did*_*joi*) . The results are shown in column (2) of Table 8, and the coefficient of the interaction term is significantly negative. This means that the more underwriting companies there are, the stronger the mitigating effect of Huimin insurance on the urban-rural consumption gap. The likely reason is that Huimin insurance programs with a larger number of participating insurers typically enjoy higher market credibility, which helps boost residents' trust in the products and thereby increases enrollment rates (70). The expansion of insurance coverage has further amplified the role of Huimin insurance in boosting rural residents' consumption and narrowing the urban-rural consumption gap. Therefore, Huimin insurance, with a larger number of participating insurers, can more effectively promote the convergence of consumption disparities.

#### 4.5.3 Heterogeneity analysis of different levels of economic development

Based on Friedman's permanent income hypothesis, residents' ability to pay for insurance and their level of demand are fundamentally constrained by their budget and income levels. There are systematic differences in the payment ability, risk perception, and actual demand for Huimin insurance among residents in regions with different levels of economic development. Therefore, this article speculates that there may be significant heterogeneity in the economic development level of the impact of Huimin Insurance on the urban-rural consumption gap. To verify this inference, this article mainly tests it from two aspects. Firstly, this article divides the research sample into first- and second-tier cities, provincial capital cities, and other cities for grouped regression analysis.^3^ The results in columns (1) and (2) of Table 9 show that the coefficient is significantly negative only in other cities. In addition, to further accurately capture the continuous impact of economic development, this article refers to the approach of Zhu et al. (71) and introduces the interaction term between policy dummy variables and per capita GDP in the benchmark regression (*did*_ln_>*per*_*gdp*). At the same time, this article divides all cities into three categories for grouped regression: per capita GDP < 50,000 yuan, 50,000–100,000 yuan, and >100,000 yuan. The results of column (3) show that the coefficient of the interaction term is significantly positive, indicating that in cities with higher levels of economic development, Huimin insurance does not narrow the urban-rural consumption gap, but rather widens it. The results of group regression also confirmed this conclusion: as shown in columns (4)–(6), the Huimin insurance only significantly narrowed the urban-rural consumption gap in low-income cities, while it actually widened the urban-rural consumption gap in high-income cities. The possible reason is that in areas with lower levels of economic development, especially rural areas, the existing medical security system still lacks sufficient coverage for major diseases and high medical expenses. Residents face higher uncertainty in medical expenses and stricter budget constraints, and their consumption decisions are more sensitive to health risk impacts (72). In this context, Huimin Insurance significantly alleviates the medical risk exposure of rural households, reduces the motivation for precautionary savings, and releases suppressed consumption demand by strengthening its protection function for major illnesses and high medical expenses, thereby promoting the convergence of the urban-rural consumption gap (46). In contrast, in highly developed economic regions, the existing medical security system has already covered basic and high medical risks to a large extent, and Huimin insurance plays a more complementary role in providing protection (73). Its marginal promotion effect on local rural residents' consumption is relatively limited; Correspondingly, the improvement of assurance certainty is more likely to translate into increased confidence among urban residents in improving their quality of life and long-term expenditures, which in turn manifests as an expansion of the urban-rural consumption gap in the short term. The above results indicate that the policy effects of Huimin insurance are more reflected in regions and groups with lower levels of economic development and relatively incomplete existing medical security systems. Its welfare provision effect is more prominent among low-income residents, further confirming the inclusive positioning of Huimin insurance characterized by “low threshold and high protection.”

**Table 9:** Heterogeneity analysis 2.

**Variables**	**(1) First and second cities Gap**	**(2) Other cities Gap**	**(3) Interaction term Gap**	**(4) Less than 50,000 Gap**	**(5) 50,000–100,000 Gap**	**(6) More than 100,000 Gap**
DID	−0.029 (−1.098)	−0.045^**^ (−2.541)	−1.121^***^ (−6.310)	−0.079^**^ (−2.510)	−0.018 (−0.914)	0.038^**^ (2.161)
did_ln_per_gdp			0.098^***^ (6.109)			
Control	YES	YES	YES	YES	YES	YES
City FE	YES	YES	YES	YES	YES	YES
Time FE	YES	YES	YES	YES	YES	YES
Constant	1.164 (0.930)	2.244^**^ (2.414)	2.728^***^ (3.686)	3.919^**^ (2.178)	−1.817^*^ (−1.671)	0.195 (0.224)
Observation	432.000	1584.000	2016.000	793.000	878.000	345.000
*r* ^2^	0.572	0.500	0.511	0.421	0.522	0.728

^***^, ^**^, and ^*^ represent significance levels of 1%, 5%, and 10%, respectively, with T-values in parentheses.

### 4.6 Analysis of impact mechanism

Based on the theoretical analysis and research hypotheses in the previous text, this article starts from the “preventive savings” channel to test the possible mechanism of the impact of Huimin insurance on the urban-rural consumption gap. Referring to the study by Zhu et al. (71), the ratio of year-end savings balance between urban and rural areas to GDP is used as a proxy variable for rural residents' precautionary savings (*Save*); According to Jiang's (57) approach to mechanism testing, the first step is to examine whether policy variables have a significant impact on mechanism variables. The results in column (1) of Table 10 show that the coefficient of the core explanatory variable is significantly negative, indicating that the Huimin insurance can effectively reduce the precautionary savings of rural residents. Furthermore, existing studies have also shown that a decrease in precautionary savings can increase current consumption tendencies and drive an increase in consumer spending, and this effect is more significant among rural residents, thereby promoting the convergence of the urban-rural consumption gap (48, 74). Overall, hypothesis H2 has been validated.

**Table 10:** Analysis of impact mechanism.

**Variables**	**(1) Preventive savings for rural residents Save**
DID	−0.014^*^ (−1.746)
Control	YES
City FE	YES
Time FE	YES
Constant	6.616^***^ (16.730)
Observation	2016.000
*r* ^2^	0.785

^***^, ^**^, and ^*^ represent significance levels of 1%, 5%, and 10%, respectively, with T-values in parentheses.

## 5 Further analysis

Benchmark regression has confirmed that Huimin Insurance can significantly narrow the urban-rural consumption gap. On this basis, a naturally derived question is how the policy affects the two major groups of urban and rural areas separately, whether it mainly increases the consumption of rural residents or also affects urban residents, but the overall gap is narrowed due to the relatively limited impact intensity. Therefore, this section decomposes this overall effect by replacing the dependent variables in the benchmark regression with the logarithm of per capita consumption expenditure of urban and rural residents for regression testing. The estimated results are shown in columns (1)–(4) of Table 11. Among them, columns (1) and (2) show that regardless of whether control variables are included or not, the impact of Huimin insurance on rural residents' consumption is significantly positive; However, columns (3) and (4) indicate that the impact of Huimin insurance on urban residents' consumption is not significant. This result further confirms the benchmark conclusion of this article: There is a more stable correlation between the promotion of Huimin insurance and the improvement of rural residents' consumption, which provides a supplementary explanation for its relationship with the narrowing of the urban-rural consumption gap.

**Table 11:** Further analysis.

**Variables**	**Rural residents' consumption expenditure**		**Urban residents' consumption expenditure**	
	**(1) Ln_r_i**	**(2) Ln_r_i**	**(3) Ln_u_i**	**(4) Ln_u_i**
DID	0.017^***^ (2.641)	0.016^***^ (2.655)	−0.001 (−0.111)	−0.003 (−0.538)
Ln_per_gdp		0.130^***^ (10.029)		0.089^***^ (7.581)
Ln_gov		0.058^***^ (3.093)		0.081^***^ (4.732)
Education		−0.086 (−0.709)		0.026 (0.242)
Finance		0.002^***^ (4.976)		0.001^**^ (2.022)
Medical		0.027 (0.747)		0.029 (0.889)
Pen		−0.113^**^ (−2.539)		0.029 (0.732)
Med		0.043 (1.267)		0.035 (1.135)
Ind		0.073^***^ (10.804)		0.019^***^ (3.095)
Inter		−0.000 (−0.419)		0.000 (0.411)
Constant	9.126^***^ (2304.340)	6.531^***^ (21.391)	9.805^***^ (2827.300)	7.488^***^ (27.100)
City FE	YES	YES	YES	YES
Time FE	YES	YES	YES	YES
Observation	2016.000	2016.000	2016.000	2016.000
*r* ^2^	0.934	0.942	0.897	0.904

^***^, ^**^, and ^*^ represent significance levels of 1%, 5%, and 10%, respectively, with T-values in parentheses.

## 6 Discussion

The empirical results of this article indicate that the promotion of Huimin Insurance, a universal supplementary medical insurance, is significantly correlated with the convergence of the macro-level urban-rural consumption gap in statistical significance. Its role is mainly reflected in the more obvious promotion effect on rural residents' consumption, while its impact on urban residents' consumption is relatively limited. Moreover, the above conclusion remains consistent under multiple robustness tests. This discovery supports the basic judgment that improving medical security can release residents' consumption and improve economic welfare by alleviating uncertainty constraints at the macro level, and also echoes the research conclusion of Zhou and Cao (75) that supplementary medical insurance has a stronger driving effect on rural consumption than urban areas. Heterogeneity analysis shows that this effect is significant in cities with relatively low levels of economic development, and is stronger in Huimin insurance products with higher levels of protection and more underwriting institutions. This indicates that the policy effects and institutional advantages of Huimin insurance are more likely to be translated into actual welfare improvements in underdeveloped areas and vulnerable groups, which is consistent with the original intention of Huimin insurance's institutional design of “wide coverage, low threshold, and affordability.” In terms of the mechanism of action, the promotion of Huimin insurance is significantly correlated with the reduction of preventive savings among rural residents, providing empirical evidence for the macro-level path of “improvement of medical security—decrease in risk expectations—adjustment of consumption decisions.”

However, there are still several shortcomings in this article that require further expansion in future research. Firstly, limited by data availability, this article identifies the average effect between the promotion of Huimin insurance and the urban-rural consumption gap based on macro data at the prefecture level city level, which makes it difficult to directly reflect the specific behavioral adjustments of households or individuals at the micro level; At the same time, the results at the macro level may be influenced to some extent by ecological fallacies and aggregation biases, especially in the analysis of the mechanism of action. This article mainly relies on preventive savings-related indicators at the macro level, and it is not yet possible to confirm whether there are changes in risk awareness and consumer decision adjustments in the same direction at the micro level. Future research can combine household survey data or microdata on medical expenditures to further examine the specific pathways through which welfare insurance affects consumption. Secondly, in terms of instrumental variable identification strategy, this article uses “last year's coverage of Huimin insurance in other cities in the same province” as the instrumental variable, which has certain explanatory power at the empirical and theoretical levels. However, its exclusivity assumption may still be challenged, especially in the context of regional economic shocks, fiscal policies, and spatial correlations in public service supply. This instrumental variable may affect the consumption of local residents through other channels. Future research can attempt to combine spatial econometric methods or introduce more institutional exogenous instrumental variables to further enhance the credibility of identification. Finally, the core policy variable of this article is the binary interaction term between the treatment group and the time dummy variable under the DID framework. Although this setting can capture the net effect of policy initiation, it fails to further distinguish the differences in policy intensity among cities in dimensions such as participation rates. Therefore, this result reflects more on the average effect of “whether to promote.” With the gradual improvement of data, future research can construct policy intensity indicators such as the coverage rate of welfare insurance.

## 7 Summary and recommendations

This article is based on panel data from 224 cities in China from 2015 to 2023, and uses a multi-period difference in differences (DID) method to examine the relationship between the promotion of Huimin insurance and the urban-rural consumption gap from a macro perspective. The research results indicate a significant statistical correlation between the implementation of Huimin insurance and the narrowing of the urban-rural consumption gap, and this conclusion remains consistent under multiple robustness tests. Further heterogeneity analysis revealed significant differences in this association across different cities and product characteristics. Specifically, the correlation effect was significant in cities with relatively lower levels of economic development, and was more significant in Huimin insurance products with higher levels of protection and more participating underwriting institutions. On this basis, mechanism analysis explored the possible pathways through which the Huimin insurance affects the urban-rural consumption gap from a macro perspective. The results indicate that there is a certain correlation between its promotion and the reduction of rural residents' precautionary savings. However, due to data hierarchy limitations, this article cannot directly identify individual-level behavioral adjustments. Further analysis shows that there is a significant correlation between Huimin insurance and the improvement of rural residents' consumption level, while the correlation between Huimin insurance and changes in urban residents' consumption is not obvious. This provides supplementary evidence at the macro level that Huimin insurance may have a stronger universal orientation.

Based on the above conclusions, this article proposes the following suggestions: firstly, we should continue to adhere to and optimize the top-level design of Huimin insurance, and adhere to its fundamental attribute of inclusiveness. On this basis, we will deepen product design, promote the upgrading of its protection content from “basic protection” to “upgraded protection,” moderately expand the coverage of special drug catalogs and advanced diagnostic technologies, and better meet the diverse protection needs of different groups. Secondly, further promote the tilt of Huimin insurance toward economically underdeveloped areas and rural areas. The results indicate that Huimin Insurance has a more significant alleviating effect on the urban-rural consumption gap in underdeveloped areas, and its promoting effect on rural residents' consumption is significantly stronger than that on urban residents. Therefore, it is recommended that local governments take the lead in exploring the establishment of a tiered premium subsidy mechanism that matches the income level of rural residents, and provide a higher proportion of financial subsidies to low-income groups to effectively reduce their participation threshold. At the same time, guide insurance institutions to develop more targeted “rural version” or “county-level exclusive” Huimin insurance products based on the characteristics of the county-level economy and disease risk structure, so as to make the protection responsibility more in line with local actual needs. Thirdly, strengthen grassroots policy promotion and service sinking. By enhancing the awareness and participation of rural residents in the Huimin insurance through multiple channels, and exploring the inclusion of insurance coverage and implementation effectiveness in the relevant assessment system for rural revitalization, a long-term mechanism that emphasizes both policy incentives and constraints will be formed. This will ensure that the Huimin insurance accurately benefits the target group while expanding the scale of insurance coverage, continuously unleashing the potential of rural consumption, and enhancing its sustainability and inclusive effect in underdeveloped areas. Fourthly, we will further integrate the Huimin insurance into the multi-level medical security system, strengthen the connection and support with basic medical insurance, promote the realization of “one-stop” settlement services, and simplify the reimbursement process for rural residents.

## Data Availability

Publicly available datasets were analyzed in this study. This data can be found here: The basic data of this article comes from the China Urban Statistical Yearbook, and the data on Huimin Insurance is manually compiled by the author. It is under consideration for further research and will not be made public for the time being. For other information, please contact the corresponding author.

## Footnotes

1 Data Source: Statistical Bulletin on the Development of China's Medical Security Industry in 2024: https://www.nhsa.gov.cn/art/2025/7/14/art_7_17248.html.

2 Data Source: Statistical Bulletin on the Development of China's Health Care Industry in 2024: https://www.nhc.gov.cn/guihuaxxs/c100133/202512/f1c3a3c617484a27a1a26a468afbaeee/files/2024%E5%B9%B4%E6%88%91%E5%9B%BD%E5%8D%AB%E7%94%9F%E5%81%A5%E5%BA%B7%E4%BA%8B%E4%B8%9A%E5%8F%91%E5%B1%95%E7%BB%9F%E8%AE%A1%E5%85%AC%E6%8A%A5-20251201161542231.pdf.

3 Regarding the criteria for dividing first- and second-tier cities, this article refers to the “2025 New First-tier City Charm Ranking” released by the New First-tier City Research Institute, a city data research think tank under the First Financial Group.
